# Bedside Ultrasound Detection of Systemic Air Embolism Secondary to Fulminant Necrotizing Enterocolitis in a Neonate With Congenital Heart Disease: A Case Report

**DOI:** 10.7759/cureus.22970

**Published:** 2022-03-08

**Authors:** Ching Y Cheong, Gene Y Ong, Yek K Chor

**Affiliations:** 1 Paediatrics, Hospital Tunku Azizah, Kuala Lumpur, MYS; 2 Paediatrics, Sarawak General Hospital, Kuching, MYS; 3 Children’s Emergency, KK Women’s and Children’s Hospital, Singapore, SGP

**Keywords:** bedside ultrasound, congenital heart disease, pediatrics, necrotizing enterocolitis, air embolism

## Abstract

Systemic air embolism is a rarely reported complication of necrotizing enterocolitis in the neonatal population. It carries significant morbidity and mortality. We report a 6-day-old, term female neonate with a duct-dependent (systemic) congenital heart disease (interrupted aorta with patent ductus arteriosus and ventricular septal defect) who presented in extremis. The neonate was successfully resuscitated, mechanically ventilated, and put on intravenous prostaglandins in paediatric intensive care unit. She clinically improved but later she developed necrotizing enterocolitis which was complicated by systemic air embolism; both of which were identified by bedside ultrasound. Her condition deteriorated and she succumbed due to these complications.

## Introduction

Systemic air embolism in neonates is a fulminant condition with significant morbidity and mortality [1,2]. It had been reported in premature infants with hyaline membrane disease on mechanical ventilation, following surgical procedures, or cardiopulmonary resuscitation [1-6]. Its occurrence as a consequence of necrotizing enterocolitis is extremely rare and the diagnosis of necrotizing enterocolitis remains challenging [1,2,5]. Hence, bedside ultrasound may be a potential clinical adjunct which can be used for early diagnosis and prompt appropriate targeted management [7-9]. In this case report, we describe a neonate with a duct-dependent congenital heart disease who presented with obstructive shock at day 6 of life and later developed necrotizing enterocolitis complicated by systemic air embolism at day 20 of life at Paediatric Intensive Care Unit. The necrotizing enterocolitis was detected using ultrasound as the presence of intramural gas within thickened bowel walls. Further bedside ultrasound surveillance revealed air bubbles along the portal vein, liver, hepatic vein, right atrium, right ventricle, and these were seen passing through the ventricular septal defect, and entering the systemic circulation (left ventricle, aorta and then to the cerebral arteries). Despite further maximal support and management, the patient succumbed.

## Case presentation

A 6-day-old, term baby girl, who was born to a mother with gestational diabetes, with a birth weight of 2.7kg, presented with inconsolable crying and lethargy for one day. There was progressive worsening of irritability, poor oral intake and rapid breathing on the day of admission. On examination, she was lethargic and dehydrated. Her blood glucose was low (0.9mmol/L), dextrose bolus and fluid resuscitation were commenced. She had a fluid-resistant shock and required intubation and multiple vasoactive agents (dopamine, dobutamine adrenaline, milrinone and noradrenaline) to maintain hemodynamic stability in the paediatric intensive care unit. Intravenous antibiotics (crystalline-penicillin and cefotaxime) were started for empiric treatment of severe neonatal sepsis while waiting for blood cultures and other septic workup. During the initial resuscitation, a detailed examination reviewed loud pansystolic murmur with radio-femoral delay and discrepancy of systolic blood pressure between upper and lower limbs of 20-25mmHg. Bedside echocardiography done by a paediatric intensivist on call (trained and highly experienced in bedside ultrasound) revealed an interrupted aortic arch type B which is a duct-dependent systemic cardiac lesion. Specifically, the bedside echocardiography of the neonate showed a discontinuity between ascending aorta and descending aorta, in which her ascending aorta gave rise to innominate and left common carotid artery and left subclavian artery and descending aorta gave rise to left subclavian artery, with a small aortic annulus. There was also a patent ductus arteriosus (3mm), and a large ventricular septal defect. Intravenous prostaglandin was started in order to keep the patent ductus arteriosus patent. The diagnosis was confirmed by a visiting paediatric cardiologist soon after.

The obstructive shock led to end-organ damage. She had acute kidney injury with oliguria, severe metabolic acidosis and hyperkalemia (serum urea 12.7 mmol/L, sodium 127 mmol/L, potassium 7.4 mmol/L, creatinine 297 µmol/L, and arterial blood gas showed pH of 7.14, pCO2 35 mmHg, pO2 88 mmHg, bicarbonate 11.8 mmol/L, base excess -16). Continuous veno-venous hemodiafiltration (CVVHDF) was commenced for a total of five days. Her condition gradually improved; inotropic support was weaned off. The neonate was thus gradually started on infant formula on day 6 of admission and able to achieve full enteral feeds by day 12 of admission. Her ventilator support was gradually tapered down to low ventilator settings (Synchronised Intermittent Mandatory Ventilation with Pressure Regulated Volume Control mode with tidal volume of 6 ml/kg, Pressure support 10 cmH2O, PEEP 7 cmH2O, ventilator rate 30/minute, FiO2 30%).

**Figure 1:**
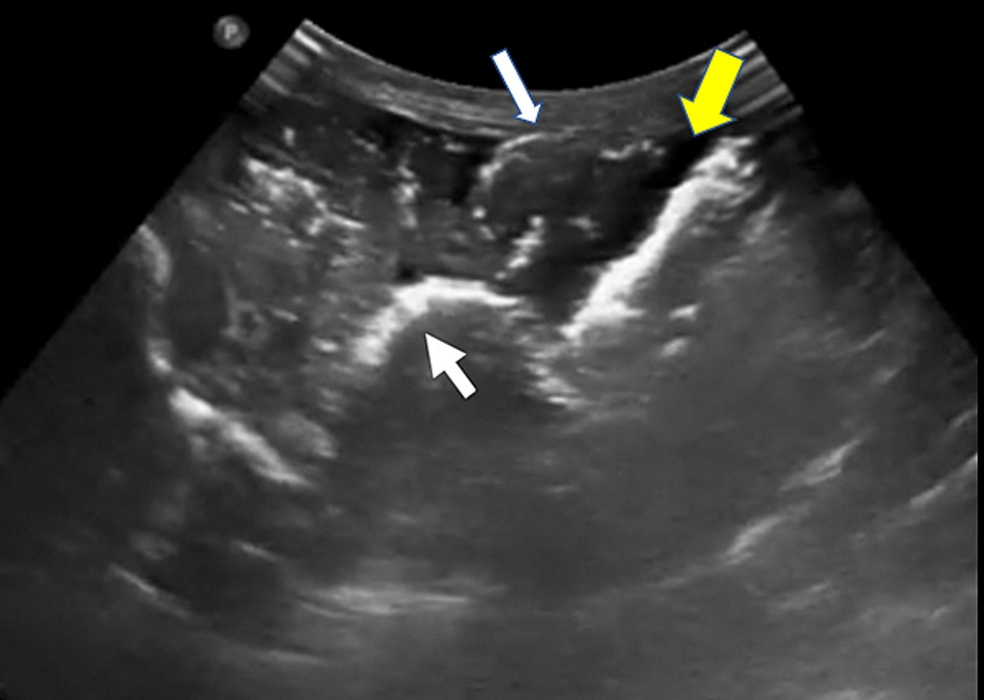
Bedside ultrasound image obtained from the lower abdomen (transverse view) using curvilinear probe, demonstrating edematous bowel wall with intra-mural air which appeared as hyper-echoic lining along the bowel wall (white arrows) suggestive of pneumatosis intestinalis. There was also free fluid demonstrated in the ultrasound image as shown by the yellow arrow. White arrows: intra-mural air which appeared as hyper-echoic lining along the bowel wall suggestive of pneumatosis intestinalis. Yellow arrow: Free fluid

However, on day 14 of admission, the neonate developed watery loose stools, followed by gastrointestinal bleeding (blood-stained nasogastric aspirates and blood in stool). Her abdomen became more distended. An urgent bedside ultrasound of the abdomen was performed by the attending paediatric intensivist which showed generalised edematous bowels with intramural air (Figure 1).

An ultrasound video clip of this is attached for reference (Video 1).

**Video 1:** Bedside ultrasound using curvilinear probe at the lower abdominal wall (Sagittal view) demonstrating oedematous bowels with intramural air (hyperechoic lining along the bowel wall) and also intra-abdominal free fluid.

**Figure 2:**
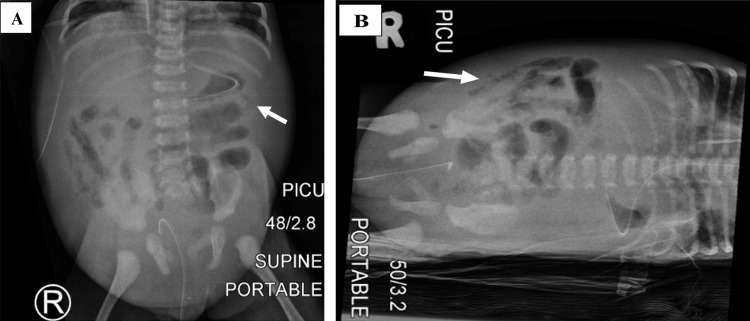
Abdominal radiographs: (A) supine and (B) left lateral decubitus radiographs showed radiologic features of pneumatosis intestinalis (white arrow). White arrow: pneumatosis intestinalis

Abdominal radiographs (Figure 2A, 2B) showed radiological features of pneumatosis intestinalis.

The diagnosis of necrotizing enterocolitis was made clinically and facilitated by bedside ultrasound and abdominal radiographs. The risk factors identified were underlying cardiac output limitation due to the underlying heart condition, prostaglandin infusion and overzealous feeding regime. She was treated with extended broad spectrum intravenous antibiotics (cefepime and amikacin) and an urgent paediatric surgical consultation was made. Her laboratory data revealed marked leucocytosis (white blood cell count of 27.8 x 10^9^/L), anemia (Haemoglobin 8.8 g/dL), thrombocytopenia (89 x 10^9^/L), deranged coagulation profile (prothrombin time 28.4 seconds, international normalized ratio 2.7, activated partial thromboplastin time 49.8 seconds). Multiple blood product transfusions (packed cell, platelet and fresh frozen plasma) were needed to stabilise her condition.

Despite resuscitation and maximal support, her condition progressed rapidly. Re-evaluations with bedside ultrasound subsequently revealed systemic air embolism, as demonstrated by widespread intra-vascular micro-bubbles entering from the portal veins, liver parenchyma, hepatic veins, right atrium, across the ventricular septal defect and eventually into the systemic circulation (Videos 2-6).

The widespread intravascular air micro-bubbles could be seen as echogenic dots on bedside ultrasound in the portal, hepatic veins, entering into the inferior vena cava and right atrium in the subcostal sagittal view in Video 2.

**Video 2:** Subcostal ultrasound image using phased array probe (sagittal view), demonstrating air embolism (hyperechoic micro-bubbles) in the circulation at portal vein, hepatic veins, inferior vena cava and right atrium.

**Figure 3:**
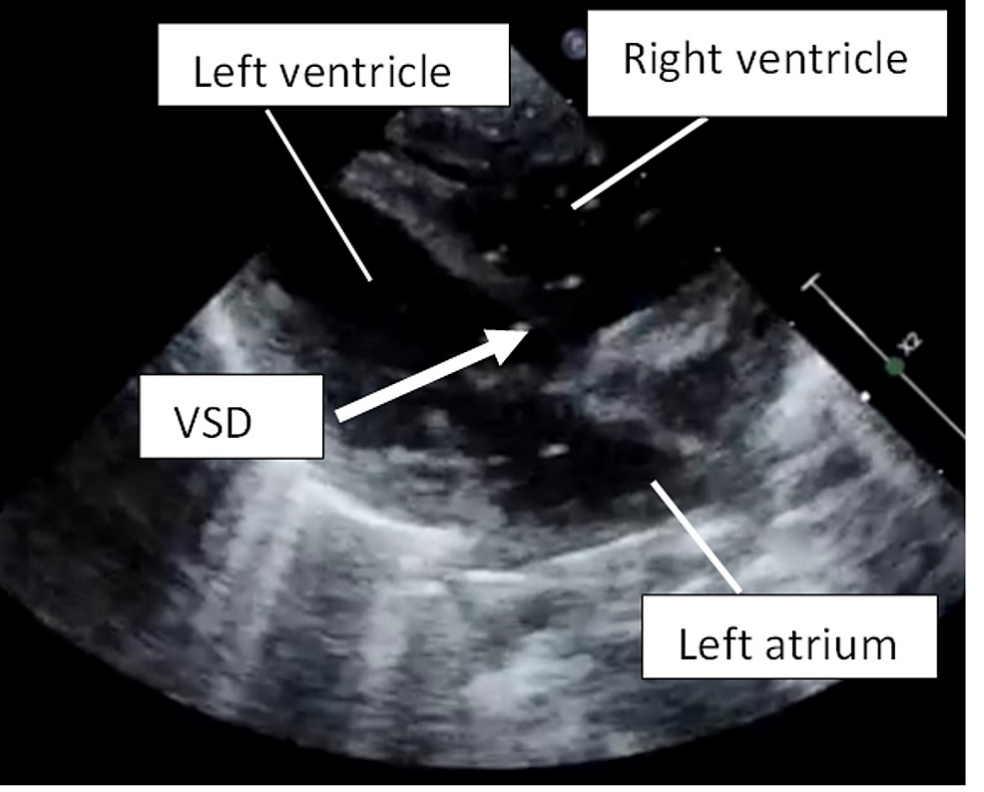
Bedside cardiac ultrasound (parasternal long axis) demonstrating air embolism (bright dots) going across the moderate ventricular septal defect (VSD). Bright dots: air embolism (air bubbles) Arrow: ventricular septal defect (VSD)

Further bedside ultrasound surveillance demonstrated that these micro-bubbles went across the ventricular septic defect (Figure 3).

Bedside cardiac ultrasound, on the parasternal long axis view, demonstrated a large ventricular septal defect and micro-bubbles (hyper-echoic “dots”) were observed going across the ventricular septal defect (Video 3).

**Video 3:** Bedside ultrasound showing air embolism going across the ventricular septal defect.

The air embolism (microbubbles) was also detected in the liver, portal vein and systemic circulation (namely inferior vena-cava and descending aorta) from the ultrasound image (Video 4).

**Video 4:** Ultrasound video clip using a phased array probe (transverse view) placed at subcostal region demonstrated air embolism in the abdominal aorta, inferior vena cava, portal vein and hepatic vessels.

As the air embolism was systemically widespread, cranial ultrasound was also performed. Air embolism (seen as microbubbles) was detected in the three major cerebral arteries namely anterior, middle and posterior cerebral arteries (Videos 5-7).

**Video 5:** Transcranial ultrasound using a phased array probe placed at anterior fontanelle (coronal view) demonstrating air embolism (moving bright dot) along the anterior cerebral artery at the region of the inter-hemispheric space. Moving bright dot: air embolism

**Video 6:** Transcranial ultrasound using phased array probe placed at the right temporal region along the squamous bone (transverse view) demonstrating a single microbubble (moving dot) travelling along the posterior cerebral artery and then into the right peduncle of the midbrain causing air embolism in the midbrain. Moving bright dot: micro-bubble (air embolism)

**Video 7:** Transcranial ultrasound using a phased array probe placed over anterior fontanelle (coronal view) demonstrating air embolism (moving bright dot) along the right middle cerebral artery territory. There was also a round hyperechoic lesion at the right thalamic lesion suggestive of either a right thalamic bleed or abscess. Moving bright dot: air embolism

Her ventilatory support was escalated to high-frequency oscillatory ventilation (with FiO2 100%) due to progressive difficulty in maintaining her saturation. She was hemodynamically labile and needed high inotropic support. Despite aggressive measures to stabilize her condition, she continued to deteriorate further. Unfortunately, she succumbed to her illness on day 21 of admission.

## Discussion

Systemic air embolism in neonates is a fulminant condition with a very low survival rate [1,2,4,10,11]. To date, it has been reported in neonates which require mechanical ventilation, especially in preterm infants with respiratory distress syndrome (as a result of barotrauma) [2,4,6,10]. High positive pressure ventilation may result in elevated trans-alveolar pressure, alveolar rupture and subsequently allowing dissection of air into pulmonary circulation and systemic circulation [2,4,6,10]. Other causes of air embolism include advanced necrotizing enterocolitis (acute mural necrosis of the bowel wall, resulting in the passage of intestinal luminal air into the vasculature) [1,5], after cardiopulmonary resuscitation [3], introduction of air via venous catheters or following surgical procedures [2,4].

For this patient, bedside ultrasound performed by the paediatric intensivist detected the necrotizing enterocolitis and subsequently systemic air embolism. The ultrasound showed bowel wall oedema with evidence of pneumatosis intestinalis, and air embolism seen as air microbubbles detected in the portal vein, liver, hepatic veins, inferior vena cava, and entering the right heart. The large ventricular septal defect resulted in paradoxical air embolism in our patient. Cerebral air embolism was detected using transcranial ultrasound. This is a fatal condition as observed in previous literature [11-15].

Pneumatosis intestinalis or intramural gas can be seen as echogenic foci along the thickened bowel wall in necrotizing enterocolitis. If they aggregate, “A-lines” may be seen posteriorly. If extensive, a “circle sign” or an echogenic ring within the thickened bowel wall may be seen on ultrasound [16]. Systemic air embolism on ultrasound may be seen as echogenic “micro-bubbles” with a “snowstorm” appearance in the blood vessels and heart. This has been used by cardiologists for diagnosis of congenital cardiac shunts using “bubble test” and also reported in systemic air embolism [17].

The likely source of air embolism in our case can be either prolonged invasive mechanical ventilation or advanced necrotizing enterocolitis. The ventilator setting for the patient was initially not high when the air embolism was observed, hence the likelihood of mechanical ventilation being the cause of air embolism was less likely. All the central venous access for the neonate had been removed after seven days post-admission. Accidental venous catheter-related air embolism was thus also unlikely. Catheter-related systemic air embolism is usually transient, not extensive nor generally widespread.

Our patient developed advanced necrotizing enterocolitis (Modified Bell stages III b) at the second week of admission. Given the low-pressure ventilatory settings prior to her clinical deterioration, lack of air leak syndromes, we hypothesised that the systemic air embolism was more likely due to the necrotizing enterocolitis. To our knowledge, there are only a few published reports on systemic air embolism in necrotizing enterocolitis [1,5] and this report would be the first to show this in “real-time” ultrasound video clips.

Bedside or point-of-care ultrasound (POCUS) has increased in popularity in recent years, especially in emergency medicine departments and intensive care units in low and middle incomes countries [7-9]. This case demonstrated that POCUS played an important role in our patient, which was not only helpful in detecting critical ductal-dependent congenital heart disease, but also detected necrotizing enterocolitis and systemic air embolism. The European Society of Paediatric and Neonatal Intensive Care (ESPNIC) recommended that POCUS should not be routinely used as a screening tool for diagnosing congenital heart diseases in critically ill neonates, unless the operator received advanced echocardiography training [8]. However, it may be important to diagnose critical duct-dependent congenital heart disease in sick neonates, especially in our patient as timely initiation of intravenous prostaglandin was live-saving by keeping the ductus arteriosus patent. ESPNIC recommended that ultrasound may be useful as an adjunct to detect necrotizing enterocolitis as radiographs may not be sufficiently sensitive, and ultrasound could provide additional information regarding bowel wall thickness, intramural gas, portal or hepatic venous gas, peritoneal fluid and as well as vascular perfusion [8]. In the extremely rare situation whereby systemic air embolism did occur, POCUS may be a potentially useful clinical adjunct to identify air embolism in the setting whereby advanced imaging techniques such as computed tomography or magnetic resonance imaging may not be easily accessible and available [7].

## Conclusions

Bedside ultrasound done by trained personnel, as illustrated in this case report, facilitated the early diagnosis and timely management of a duct-dependent congenital heart lesion (interrupted aortic arch), and subsequent pneumatosis intestinalis in necrotizing enterocolitis which was complicated by systemic air embolism, an extremely rare and lethal condition. This case report aimed to demonstrate and share the rare imaging findings of systemic air embolism using ultrasound. Further studies are needed to evaluate its clinical value in this condition in terms of training required for diagnosis, as well as its potential for clinical prognosis.
